# Hepatitis B virus promotes cancer cell migration by downregulating miR-340-5p expression to induce STAT3 overexpression

**DOI:** 10.1186/s13578-017-0144-8

**Published:** 2017-04-12

**Authors:** Qiushuang Xiong, Shaoshuai Wu, Jingwen Wang, Xianhuang Zeng, Jianwen Chen, Mingcong Wei, Haotong Guan, Chengpeng Fan, Lang Chen, Deyin Guo, Guihong Sun

**Affiliations:** 1 School of Basic Medical Sciences, Wuhan University, Wuhan, 430072 People’s Republic of China; 2 Hubei Province Key Laboratory of Allergy and Immunology, Wuhan, People’s Republic of China

**Keywords:** miR-340-5p, HBV–HCC, STAT3, Cell migration

## Abstract

**Background:**

Hepatocellular carcinoma (HCC) is the third leading cause of cancer-related deaths worldwide, and infection with hepatitis B virus (HBV) is a leading cause of HCC. Previous studies have demonstrated that expression of the tumor inhibitor miR-340 is significantly downregulated in HCC tissues compared with normal liver tissues. However, the precise biological role of miR-340-5p in HBV–HCC and its molecular mechanism of action remain unknown.

**Results:**

Expression of miR-340-5p was downregulated in HBV-associated HCC liver tissue and HBV-infected cells, facilitating migration of liver cancer cells. Signal transducer and activator of transcription (STAT)3 was found to be a direct functional target of miR-340-5p. The regulation of STAT3 expression by miR-340-5p was assessed using qRT-PCR and western blotting, and the effects of exogenous miR-340-5p and STAT3 on the migration of HBV-infected cells were evaluated in vitro using Transwell^®^ and wound-healing assays. The expression of E-cadherin and vimentin, associated with epithelial–mesenchymal transition, was also assessed using Western blotting after transfection of miR-340-5p mimics and/or STAT3 expression vectors. Overexpression of STAT3 resulted in rescue of HBV effects, decreased E-cadherin expression, increased vimentin expression, and ultimately, enhanced cell migration. Re-introduction of the STAT3 CDS led to marked reversal of the inhibition of cell migration in HBV-infected cells mediated by miR-340-5p.

**Conclusions:**

Hepatitis B virus promotes the migration of liver cancer cells by downregulating miR-340-5p expression to induce STAT3 overexpression. Our results show that STAT3 plays a key role in regulating cell migration in HBV–HCC involving miR-340-5p.

## Background

Hepatocellular carcinoma (HCC) is the sixth most common malignancy worldwide. Both the incidence and mortality rates of HCC continue to increase. Population-based studies have shown that HCC is the third leading cause of cancer-related deaths worldwide [1]. The development of HCC is believed to be most closely associated with hepatitis B virus (HBV) or hepatitis C virus (HCV) infection [2]. HBV is an enveloped, partially double-stranded DNA virus with a genome size of 3.2 kb. A previous study demonstrated that HBV infection accounts for >60% of all liver cancers in the developing countries but <25% of cases in the developed countries [2]. The binding of the HBV to its receptor leads to the activation of numerous signaling pathways that regulate important biological functions, such as inflammatory and immune responses, as well as tumorigenesis. HCC initiation and progression are currently thought to involve epithelial–mesenchymal transition (EMT) [3], in which epithelial cells acquire mesenchymal features and increased motility and invasiveness [4, 5]. EMT is characterized by reduced intercellular adhesion, loss of epithelial markers (such as E-cadherin), and acquisition of mesenchymal markers [including vimentin, matrix-degrading proteases [e.g., matrix metalloproteinases (MMPs)], and N-cadherin [6]. The EMT of cancer cells enables them to leave the primary tumor site and migrate to and invade surrounding and distant tissues/organs. However, obtaining a comprehensive understanding of the underlying mechanism by which HBV promotes tumor cell migration requires further elucidation.

EMT involves a number of molecular processes, including the activation of transcription factors, changes in the expression levels of specific cell surface and cytoskeletal proteins, and the production of various extracellular matrix-degrading enzymes. Accumulating evidence suggests that certain microRNAs (miRNAs) play important roles in EMT. miRNAs are endogenous, evolutionarily conserved, small noncoding RNA molecules (18–25 nucleotides in length) that negatively regulate the expression of numerous target genes at the posttranscriptional level through sequence-dependent translational repression and/or target mRNA degradation [7]. Recent studies have revealed that miRNA-340 (miR-340) expression is downregulated in several types of cancer, demonstrating that miR-340 can act as a tumor suppressor and is, thus, involved in cell differentiation, proliferation, apoptosis, and tumor cell migration and invasion [8–11]. In addition, a previous study indicated that miR-340 represses the proliferation, migration, and invasion of HCC cells by targeting the Janus kinase (JAK) via the JAK1/signal transducer and activator of transcription (STAT)3 signaling pathway [12]. However, the underlying mechanism of miR-340 in HBV-associated HCC remains unclear.

STAT3 is a latent transcription factor that is activated in response to cytokine-induced stimuli. After cytokine binding, the receptors are quickly phosphorylated by JAK kinases. STAT3 is then phosphorylated by JAK kinase, after which STAT3 dimerizes and is translocated into the nucleus, where it regulates the expression of target genes [13]. STAT3 is constitutively activated in various cancers, including HCC [14, 15]. Activated STAT3 contributes to oncogenesis by promoting cell proliferation, preventing apoptosis, and impairing host anti-tumor immune responses [16, 17]. Knockdown of the STAT3 protein via STAT3 antisense oligonucleotide was shown to markedly inhibit the proliferation and tumorigenic growth of an HCC cell line transplanted into nude mice [18]. Constitutive activation of STAT3 plays a pivotal role in the development of many types of human tumors [19], and increased levels of STAT3 protein are found in many human tumors, including HCC [14, 16, 20]. An increase in the level of un-phosphorylated STAT3 contributes greatly to the development of cancers by driving EMT. However, the role and mechanism of the upregulation of total STAT3 in tumorigenesis and cancer progression remain unclear. Our study shows that HBV regulates the STAT3 signaling pathway via miR-340-5p to facilitate cancer cell migration. In the present study, we sought to gain insights into the regulation of miR-340-5p in HBV-associated HCC. Our findings indicate that miR-340-5p directly regulates STAT3 in HBV-mediated promotion of hepatoma cell migration. Our data, thus, provide new insights into the mechanism through which HBV promotes hepatoma cell migration.

## Methods

### Clinical specimens

Clinical HCC specimens were obtained from Zhongnan Hospital of Wuhan University. The specimens were verified and classified according to the World Health Organization Classification of Tumors guidelines by two experienced clinical pathologists. Clinical samples were collected from patients after obtaining informed consent in accordance with a protocol approved by the Ethics Committee of Wuhan University (Wuhan, China).

### Reagents and cell culture

HepG2, HepG2.2.15, HuH7, and HEK293T cells were obtained from the Type Culture Collection of the Chinese Academy of Sciences (Shanghai, China). All cells were cultured in Dulbecco’s modified Eagle’s medium (Gibco/Life Technologies, US) supplemented with 10% fetal bovine serum (FBS; Gibco/Life Technologies) as a complete growth medium and maintained at 37 °C with 5% CO_2_ in a humidified chamber. The miR-340-5p mimics, inhibitors, corresponding controls, and siSTAT3 RNA sequence were designed and synthesized by RiboBio (Guangzhou, China). Cell transfection and co-transfection were performed using Lipofectamine 2000 when the cells reached 70% confluence. Transfection efficiency was verified by quantitative real-time PCR. After 48 h, the cells were harvested for further analysis.

### RNA extraction and quantitative real-time PCR

Total RNA was extracted using TRIzol^®^ reagent (Life Technologies) according to the manufacturer’s protocol. One microgram of total RNA was reverse transcribed into cDNA using a FastQuant RT kit (Tiangen Biotech, Beijing, China). Quantitative analysis of miR-340-5p was performed using Bulge-LoopTM miRNA quantitative RT-PCR primers (RiboBio) and SYBR Select Master Mix (Life Technologies) on an ABI Prism 7500 real-time PCR system (Applied Biosystems, Foster City, CA, USA). Quantitative analysis of STAT3 was performed using designed qPCR primers (Table 1) and the same SYBR Select Master Mix used for miR-340-5p qPCR. U6 snRNA was used as an internal control for miRNA analysis, and glyceraldehyde-3-phosphate dehydrogenase (GAPDH) mRNA was used as an internal control for quantitative analysis of mRNA. The results were calculated using the 2^−ΔΔCT^ method.

**Table 1:** Primers used in this study

Gene	Primer sequences (5′–3′) forward (F), reverse (R)	GenBank accession number	Product length(bp)
GAPDH	F: GAGAAGGCTGGGGCTCATTT	NM_001289745	156
	R: TAAGCAGTTGGTGGTGCAGG		
STAT3	F: GAGGACTGAGCATCGAGCA	NM_139276	85
	R: CATGTGATCTGACACCCTGAA		
STAT3-3′UTR	F: ATCCGCTCGAGATGGCCCAATGGAATC	NM_139276	2435
	R: CGACGCGTGAGGTCAACTCCATGTCAAAG		
STAT3-3′UTR-WT1+WT2	F: GCGCGCGCGGACTAGT**TTTATAA**ATAGACTTATTTTCCT	NM_139276	1191
	R: CGACGCGT**TTATAAA**CCACCTTATAGGTAGGTAAGC		
STAT3-3′UTR-MUT1	F: GCGCGCGCGGACTAGT**T** *CCGAT* **A**ATAGACTTATTTTCCT	NM_139276	1191
	R: CGACGCGTTTATAAACCACCTTATAGGTAGGTAAGC		
STAT3-3′UTR-MUT2	F: GCGCGCGCGGACTAGT**TTTATAA**ATAGACTTATTTTCCT	NM_139276	1191
	R: CGACGCGT*AATAT* **AA**CCACCTTATAGGTAGGTAAGC		
STAT3-3′UTR-MUT1+MUT2	F: GCGCGCGCGGACTAGT**T** *CCGAT* **A**ATAGACTTATTTTCCT	NM_139276	1191
	R: CGACGCGT*AATAT* **AA**CCACCTTATAGGTAGGTAAGC		

*WT* wild type, *MUT* mutation

### Luciferase reporter assay

The putative binding sites of the 3′-untranslated region (UTR) of the human genes for miR-340-5p targeting were predicted using the TargetScan Human computational methods (http://www.targetscan.org/). The partial 3′-UTR fragment of STAT3, the wild STAT3 including predicted binding sites, and the mutant STAT3 inserts with an opposite mutation in the miRNA seed sequence binding sites, were cloned into the pMIR-REPORT™ miRNA Expression Reporter Vector (Applied Biosystems). All primers were designed as shown in Table 1. HEK293T cells were seeded in triplicate at a density of 2 × 10^5^ cells per 24-well plate. Luciferase activity was monitored using the Luciferase Assay Kit (Promega) and normalized to Renilla luciferase activity, as previously described [21].

### Wound-healing assay

Cells were transfected for 24 h in 12-well plates, and upon reaching ~90% confluency, the cell monolayer was scratched with a 10-μL sterile pipette tip and then gently washed three times with phosphate-buffered saline (PBS), after which culture medium containing 2.5% FBS was added. Images of the cells along the scrape line were captured under a microscope at 0, 24, and 48 h. The wound-healing capacity was determined by measuring the change in the size of the scraped area, resulting from cell migration, using ImageJ software.

### Cell migration assays

Cell migration assays were performed using Transwell chambers measuring 6.5 mm in diameter (8-μm pore size, Corning). After transfection for 24 h, a total of 5 × 10^4^ transfected cells in FBS-free medium were seeded in the upper chamber of an uncoated Transwell chamber for the migration assay. Medium, containing 10% FBS, was then added to the lower chamber. After 24 h, cells that did not migrate were removed using a cotton swab. Cells that migrated to the lower chamber were fixed in 4% paraformaldehyde for 15 min, stained with 0.1% crystal violet (Sigma-Aldrich, St. Louis, MO, USA) for 15 min, rinsed with PBS, and then counted under a microscope. Five random views were used to count the cells.

### Western blotting analysis

Total cells or tissues were extracted using cell lysis buffer followed by immunoblotting with anti-STAT3 (Proteintech, Wuhan, China), anti-E-cadherin, anti-vimentin and anti-β-actin (Santa Cruz Biotechnology, Santa Cruz, CA, USA), anti-p-STAT3 and anti-GAPDH (Cell Signaling Technology) antibodies, as described previously [21].

### Statistical analysis

All data are expressed as the mean ± standard deviation (SD) (n = 3). Statistical analyses were performed using SPSS software, version 16.0 (SPSS, Chicago, IL, USA). Differences between two groups were analyzed using the unpaired Student’s t test, and differences between multiple groups were evaluated using one-way analysis of variance. *P* < 0.05 was considered indicative of statistical significance.

## Results

### HBV attenuates the expression of miR-340-5p both in vivo and in vitro

**Fig. 1:**
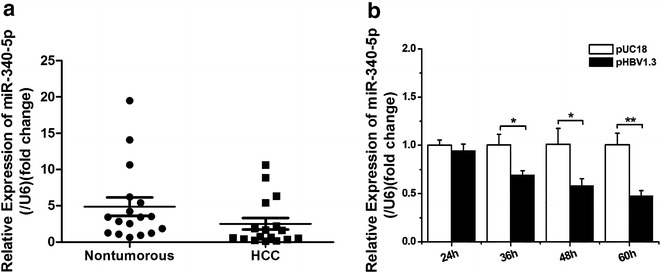
miR-340-5p is downregulated in HCC tissues and in live cancer cells. **a** Quantitative PCR for comparing the expression levels of miR-340-5p in samples from 25 paired clinical cancer cases. **b** Quantitative PCR for determining miR-340-5p expression levels in lines of HBV-infected cancer cells. Data represent mean ± SD of three independent experiments. *P < 0.05; **P < 0.01

Previous studies reported that miR-340-5p expression is downregulated in cancer tissues [9, 12]. To determine whether miR-340-5p is differentially expressed in HCC cells, para-cancerous tissues from clinical HBV samples were analyzed using qRT-PCR. The expression of miR-340-5p was significantly lower in HCC tissues than in corresponding non-cancerous liver tissue (Fig. 1a). Patients infected with HBV account for 80% of all HCC cases, and the clinical samples used in the present study were confirmed as originating from HBV-infected patients. However, in order to further confirm whether HBV plays a role in downregulating miR-340-5p expression, plasmids encoding HBV genes or control plasmids were transfected into HepG2 cells, with miR-340-5p expression monitored by qRT-PCR. The expression of miR-340-5p was markedly lower in cells transfected with the plasmid encoding HBV genes (Fig. 1b). These results indicate that HBV downregulates miR-340-5p expression both in vivo and in vitro.

### miR-340-5p inhibits the migration of hepatoma cells

**Fig. 2:**
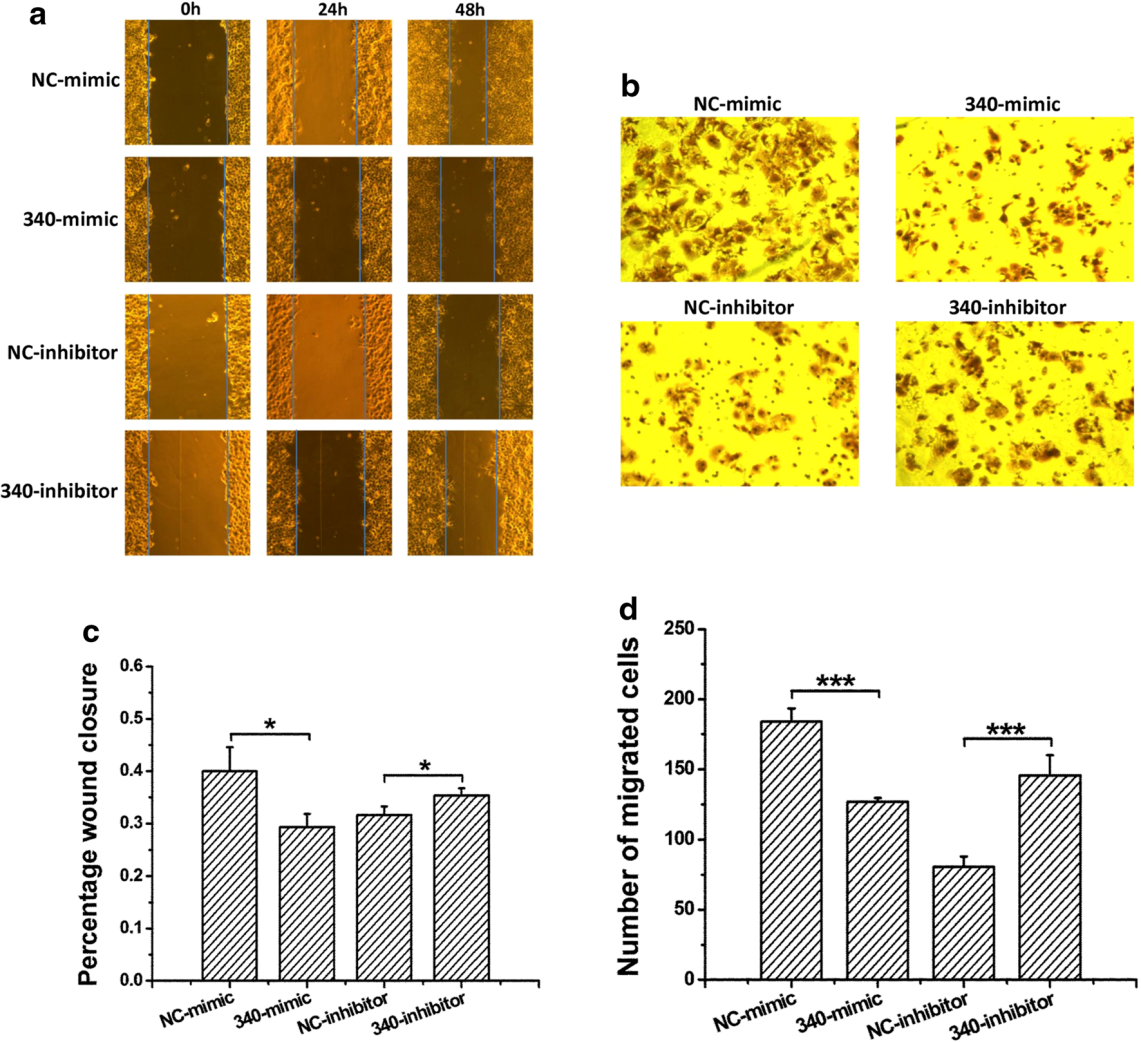
miR-340-5p inhibits the migration of HepG2 cells. **a** Representative photographs of wound-healing assays show that miR-340-5p mimics decreased wound-healing ability. NC-mimic is the negative control of miRNA mimic composed of mature miRNA double strands, and NC-inhibitor is the negative control of miRNA inhibitor containing a chemically modified mature miRNA complementary single strand. **b** Statistical analysis of the cell migration area, as measured using Image J software, demonstrated a significant reduction in cell migration caused by miR-340-5p mimics and an increment caused by miR-340-5p inhibitors. **c** Representative photographs of Transwell migration assays. **d** Statistical analysis of the results indicated that treatment with miR-340-5p mimics or inhibitors led to a dramatic decrease or increase in cell migration when compared with the corresponding negative control. ***P < 0.001

Zhang et al. suggested that miR-340 inhibits both cancer cell migration and invasion [11, 12]. In order to confirm whether downregulated expression of miR-340-5p is associated with enhanced migration capability of hepatoma cells, we performed the wound-healing and modified Boyden’s chamber assays. The results showed that miR-340-5p does indeed affect the migration of hepatoma cells. As shown in Fig. 2a, b, overexpression of miR-340-5p (340-mimic) resulted in marked inhibition of HuH7 cell migration relative to NC-mimic (NC-mimic is the negative control of miRNA mimic composed of mature miRNA double strands). And inhibition of miR-340-5p (340-inhibitor) could promote HuH7 cell migration relative to NC-inhibitor (NC-inhibitor is the negative control of miRNA inhibitor containing a chemically modified mature miRNA complementary single strand). The results of the Boyden’s chamber migration assay (Fig. 2c, d) were consistent with those of simultaneous wound-healing assays.

### miR-340-5p downregulates STAT3 expression by directly targeting the STAT3 3′-UTR

**Fig. 3:**
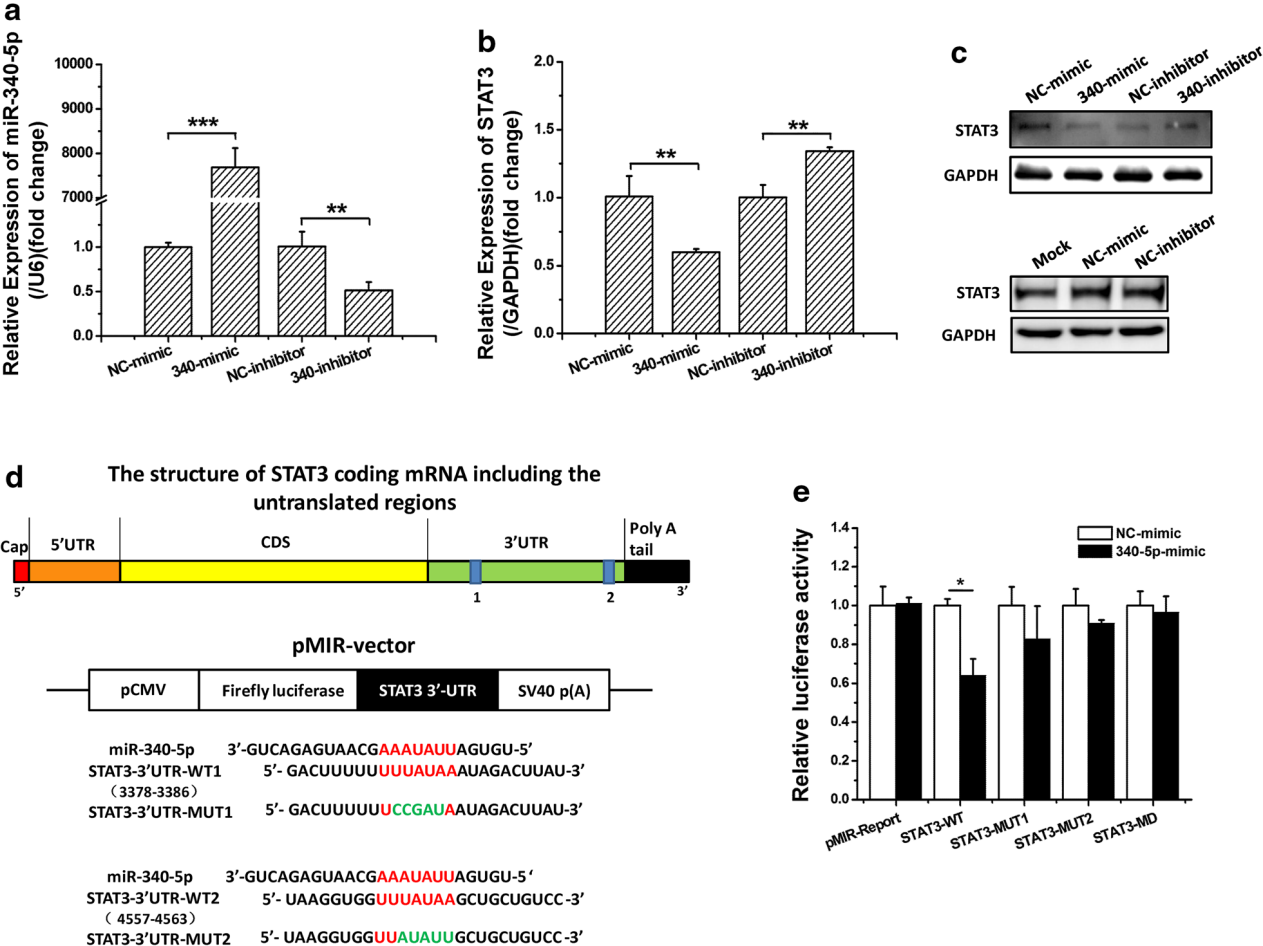
The STAT3 gene is targeted by miR-340-5p. **a**–**c** HepG2 cells were transfected with miR-340-5p mimics/inhibitors or negative control miRNA. At 48 h after transfection, total protein was extracted and analyzed by Western blotting. GAPDH was used as a loading control. **d** Sequence of the miR-340-5p binding site in the STAT3 3′-UTR was predicted using bioinformatics methods. **e** The results of 3′-UTR luciferase assays. Normalized luciferase activity, in cells transfected with reporter vector encoding the mutant-type 3′-UTR of STAT3, was not affected by overexpression of miR-340-5p compared with cells transfected with the wild-type 3′-UTR. Relative luciferase expression is shown as the mean ± standard deviation (SD). Three independent experiments were performed, and representative data are shown. *P < 0.05

To elucidate the mechanism whereby miR-340-5p inhibits the migration of human HCC cells, bioinformatics approaches were used to search for potential targets of miR-340-5p. All four bioinformatics algorithms employed (miRanda, TargetScan, miRBase, and PicTar) indicated that STAT3 was a target of miR-340-5p. In addition, high expression of miR-340-5p (Fig. 3a) dramatically suppressed the endogenous expression of STAT3 mRNA and protein in human HCC cell lines (Fig. 3b, c). In our experiment, we used the recommended dose of inhibitor which is twice the dose of mimic, and comparing with NC-mimic did, NC-inhibitor caused a significant reduction on STAT3 protein level (Fig. 3c up), which is similar to its effect on cell migration (Fig. 2d), and it’s possibly relative to the dose of transfection. So we also used the same dose of NC-mimic and NC-inhibitor to explore whether the different NC have effect on STAT3 protein expression. The result showed that the expression of STAT3 did not have significant difference with the same transfection dose of NC-mimic and NC-inhibitor (Fig. 3c below). To determine whether STAT3 is directly targeted by miR-340-5p at its 3′-UTR, luciferase reporter plasmids, containing 3′-UTR fragments of STAT3, were co-transfected into cells with 340-mimic and NC-mimic. There are two predicted miR-340-5p binding sites in the 3′-UTR of STAT3, so we constructed different 3′-UTR fragments, including wild-type (STAT3-WT) and mutant-type 3′-UTRs (STAT3-MUT), to identify the functional site (Fig. 3d). As shown in Fig. 3e, the relative luciferase activity was markedly reduced by miR-340-5p in the presence of the WT-UTR of STAT3. This reduction was determined to be sequence-specific, as the relative luciferase activity in the presence of the mutant-type UTR did not drop as sharply as in the presence of the WT-UTR. These data indicate that miR-340-5p directly targets the STAT3 3′-UTR, thereby downregulating STAT3 expression.

### miR-340-5p regulates the migration of Huh7 cells via targeting of STAT3

**Fig. 4:**
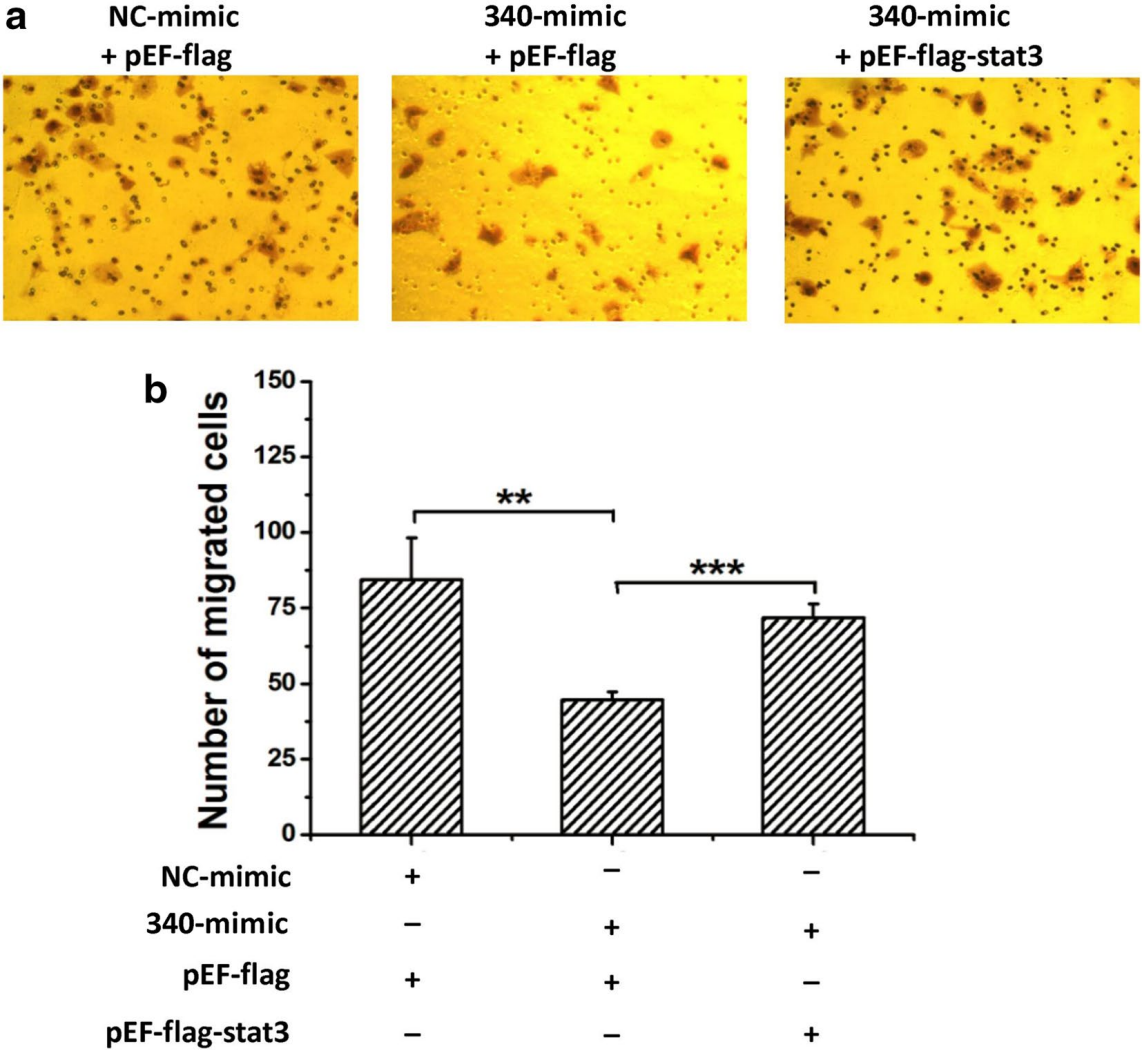
STAT3 has the opposite effect on the migration of HepG2 cells, relative to miR-340-5p. **a** Representative photographs of wound-healing assays show that miR-340-5p mimics (340-mimic) decreased wound-healing ability, and the forced expression of STAT3 reversed miR-340-5p-decreased wound-healing ability. **b** Statistical analysis of the cell migration area, as measured using Image J software. The results illustrated are the average value of three independent tests. **P < 0.01; ***P < 0.001

STAT3 was identified as a target gene of miR-340-5p. As STAT3 reportedly induces the migration of hepatoma cells [15], we sought to confirm that miR-340-5p inhibits the migration of hepatoma cells by directly regulating STAT3. Changes in the migration capability of HepG2 cells, resulting from miR-340-5p targeting of STAT3, were examined by overexpressing STAT3 using the pEF-flag-STAT3 plasmid. The results of modified Boyden’s chamber assays showed that the overexpression of STAT3 reversed the miR-340-5p-mediated inhibition of HepG2 cell migration following transient transfection with the pEF-flag-STAT3 plasmid (Fig. 4a, b). Therefore, our findings suggest that miR-340-5p inhibits the migration of hepatoma cells via targeting of the STAT3 gene. Thus, we postulate that miR-340-5p exerts a negative effect on the migration of HuH7 cells by negatively regulating STAT3, which would normally induce the migration of HuH7 cells.

### HBV promotes cell migration via downregulation of miR-340-5p expression to induce STAT3 expression

**Fig. 5:**
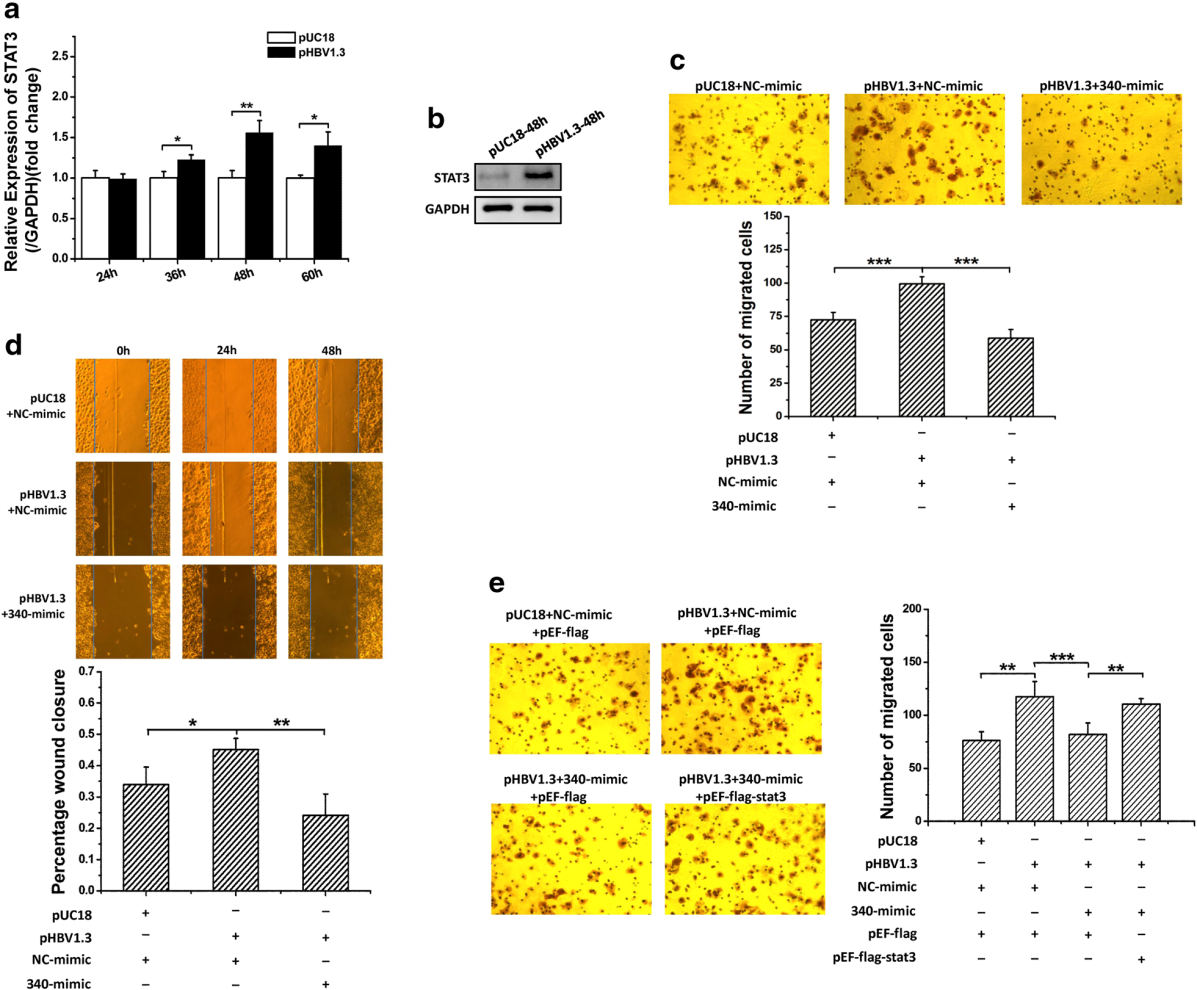
HBV induces the migration of cancer cells by inducing STAT3 overexpression via downregulation of miR-340-5p expression. **a** qRT-PCR analysis of the effect of HBV on STAT3 mRNA expression. **b** Western blot assays show HBV induces high levels of STAT3 expression. **d** Wound-healing assays show that the forced expression of miR-340-5p mimics rescue HBV-induced wound-healing ability. **c**, **e** Representative photographs of Transwell assays: **d** miR-340-5p mimics rescued HBV-induced cell migration. **e** Over expression of STAT3 rescued miR-340-5p mimics inhibition and led to a dramatic increase in cell migration. *P < 0.05; **P < 0.01; ***P < 0.001

We demonstrated that HBV downregulates the expression of miR-340-5p. Overexpression of miR-340-5p in HepG2 cells significantly suppressed cell migration, whereas co-expression of STAT3 via transfection of the cells with pEF-flag-STAT3 rescued miR-340-5p-mediated suppression. We, therefore, sought to determine whether HBV promotes cell migration via downregulation of miR-340-5p expression to induce STAT3 expression. First, we transfected cells with HBV plasmid or control plasmid. The results of qPCR and western blot analyses showed that HBV-induced STAT3 expression (Fig. 5a, b). Next, HepG2 cells were transfected with pHBV1.3 or control together with miR-340-5p mimics and overexpression STAT3 plasmids. Wound-healing assay results showed that HBV-induced cell migration was inhibited by miR-340-5p mimics (Fig. 5c). However, transwell assays indicated that overexpression of miR-340-5p inhibited cells migration that were induced by HBV; and co-transfection with STAT3 rescued the inhibition mediated by the miR-340-5p mimics (Fig. 5d, e). These results indicate that HBV induces cell migration via downregulation of miR-340-5p, resulting in overexpression of STAT3.

### Molecular mechanism through which HBV induces the migration of HuH7 cells

**Fig. 6:**
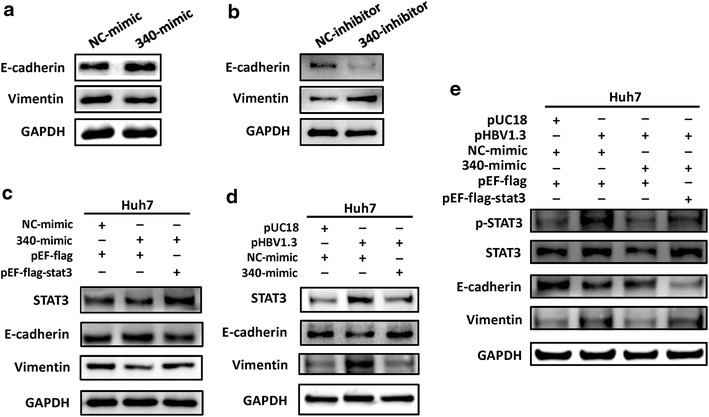
Detection of E-cadherin and vimentin expression by Western blotting. **a** Cells transfected with miR-340-5p mimics show a significant increase in E-cadherin protein expression and a dramatic decrease in vimentin protein expression. **b** Transfection with miR-340-5p inhibitors had an opposite effect (vimentin expression increased and E-cadherin expression decreased). **c** STAT3 rescued the effect of miR-340-5p mimics on vimentin and E-cadherin. **d** miR-340-5p mimics rescued the effect of HBV on vimentin and E-cadherin. **e** Expression levels of STAT3, pSTAT3 and proteins involved in migration regulated by transfecting with pHBV1.3 and/or miR-340 and/or pEF-flag-STAT3 in HuH7 cells analyzed by Western blot

We next sought to elucidate the mechanism whereby HBV induces the migration of HuH7 cells through miR-340-5p downregulation. The cell migration changes usually by EMT variation. The EMT process plays an important role in morphogenesis and cancer progression. To determine whether EMT is aberrantly changed, we evaluated the expression of E-cadherin (a protein that is a well-established epithelial cell marker), and vimentin (mesenchymal cell markers), in transfected HuH7 cells used in the previous functional study. Forced expression of miR-340-5p led to upregulation of vimentin expression and downregulation of E-cadherin expression (Fig. 6a). Correspondingly, miR-340-5p inhibition and overexpression of STAT3 had the opposite effects on E-cadherin and vimentin expression: E-cadherin levels increased and vimentin levels decreased (Fig. 6b). Forced expression of miR-340-5p rescued EMT in HBV-transfected cells (Fig. 6d). And further experiment coincided with the results that overexpression of miR-340-5p rescued the effects on E-cadherin and vimentin expression induced by HBV, and which were reversed by STAT3 (Fig. 6e). Apart from this, we wondered what is the degree of STAT3 activation in these processes, so the changes of STAT3 and p-STAT3 were also examined. As shown in Fig. 6e, after being co-transfected with DNAs or microRNAs as indicated, the level of p-STAT3 in HuH7 cells followed the same trends as the STAT3. Collectively, these analyses showed that the intrinsic mechanism, through which HBV promotes cell migration by downregulating miR-340-5p expression to induce STAT3 expression, occurs during activation of the EMT process.

## Discussion

Hepatocellular carcinoma is the most common type of primary liver cancer and is the third leading cause of cancer-related deaths [22]. HBV is one of the most important causes of HCC. Recent studies have shown that miRNAs play a fundamental role in HCC, thereby opening an avenue for novel investigations of the molecular mechanisms of HBV-HCC pathogenesis. A number of studies have shown that miR-340 acts as a tumor suppressor in several cancers, including neurofibromatosis type 1, breast cancer, and HCC [10, 11, 23, 24]. Consistent with previous studies, our data indicate that miR-340-5p expression is downregulated in HCC tissues, and importantly, the HCC tissues that we used in the present study were derived from an HBV-infected patient. Our results suggest that a decline in miR-340-5p level is closely linked to HBV-associated liver carcinogenesis. Through in vitro experiments, we verified that the downregulation of miR-340-5p expression is mediated by HBV. And, the significant effects of HBV on miR-340-5p in HBV-associated liver cancer require further investigation.

Metastasis of tumors represents the most life-threatening stage for patients with cancer. Metastasis involves a complex cascade of events leading to detachment of cancer cells from the original tumor because of altered adhesion properties and establishment of new, distant tumors. Other cellular changes promote migration, local invasion, proteolysis, angiogenesis, and survival of the tumor cells in the circulatory system. Of these events, the migration of cancer cells into surrounding tissues is a crucial early step. miRNAs are an important group of regulators of the EMT process. The overexpression of miR-340 suppresses cell migration, invasion, and metastasis in these cancers [10]. It has been reported that miR-340 suppresses the proliferation of, and invasion by, HCC cells via the regulation of JAK1 [12]. JAK1 activates STAT proteins in response to various cytokines and growth factors. The JAK1/STAT3 pathway, which is generally regarded as one of the most active signaling pathways, plays an important role in the process of malignant transformation. Recent studies have also shown that miR-340 can function as a tumor metastasis suppressor, inhibiting the metastasis of multiple malignant human tumors, such as melanoma [12], breast cancer [13], colorectal cancer [14], and liver cancer. Our results showed that overexpression of miR-340 inhibits the migration of HCC cells. We also demonstrated that STAT3 is a direct target of miR-340. In our study, miR-340-5p inhibited cell migration induced by HBV. Additional analysis confirmed that STAT3 rescues the miR-340-5p-mediated inhibition of HBV-induced migration of HCC cells, which is an important step in hepatocarcinogenesis. Our findings, showing that increased STAT3 expression, resulting from downregulation of the migration inhibitor miR-340-5p, thus provide a theoretical basis for the development of novel clinical treatments of HBV-associated liver cancer.

## Conclusions

HBV promotes the migration of liver cancer cells by downregulating miR-340-5p expression to induce STAT3 overexpression. Our results show that STAT3 plays a key role in regulating cell migration in HBV-HCC involving miR-340-5p.

## Abbreviations

HCC: hepatocellular carcinoma
HBV: hepatitis B virus
STAT: signal transducer and activator of transcription
EMT: epithelial–mesenchymal transition
miRNAs: microRNAs
miR-340: miRNA-340
JAK: Janus kinase
WT: wild-type
MUT: mutant-type
340-mimics: miR-340-5pmimics
340-inhibitor: miR-340-5p inhibitor
